# How do non-geneticist physicians deal with genetic tests? A qualitative analysis

**DOI:** 10.1038/s41431-021-00884-z

**Published:** 2021-04-28

**Authors:** Laurent Pasquier, Guy Minguet, Sylvie Moisdon-Chataigner, Pascal Jarno, Philippe Denizeau, Ginette Volf, Sylvie Odent, Grégoire Moutel

**Affiliations:** 1 Service de Génétique Clinique, Centre Référence “Déficiences Intellectuelles de causes rares” (CRDI), Centre Hospitalier Universitaire Rennes, 16 boulevard de Bulgarie, F-35203 Rennes, France; 2 INSERM U1086, Anticipe, Normandie Université, 3 avenue du Général Harris, F-14076 Caen, France; 3 Institut Mines Télécom Atlantique, Département Sciences Sociales et de Gestion, 4 rue Alfred Kastler, F-44307 Nantes Cedex 3, France; 4 Laboratoire IODE, Faculté de Droit, Université Rennes 1, 9 Rue Jean Macé, CS 54203 Rennes, France; 5 Service de Santé Publique, Centre Hospitalier Universitaire Rennes, F-35203 Rennes, France; 6 Service de Génétique Clinique, Centre Référence Anomalies du Développement CLAD Ouest, Univ Rennes, CNRS, IGDR (Institut de génétique et développement de Rennes)—UMR 6290, Centre Hospitalier Universitaire Rennes, 16 boulevard de Bulgarie, F-35203 Rennes, France; 7 Délégation Régionale Bretagne, Alliance Maladies Rares, Rennes, France; 8 Espace régional de Réflexion Ethique, Médecine légale et droit de la santé, Centre hospitalo-universitaire de Caen, Avenue de la Côte de Nacre, F-14033 Caen, France

**Keywords:** Ethics, Genetics research, Genetic testing

## Abstract

Genetic testing is accepted to be a common practice in many medical specialties. These genetic tests raise issues such as respect for basic rights, how to handle results and uncertainty and how to balance concerns for medical confidentiality with the rights of third parties. Physicians need help to deal with the rapid development of genomic medicine as most of them have received no specific training on the medical, ethical, and social issues involved. Analyzing how these professionals integrate genetic testing into the patient-provider relationship is essential to paving the way for a better use of genomics by all. We conducted a qualitative study comprising a series of focus groups with 21 neurologists and endocrinologists about their genetic testing practices in the western part of France. The interviews were transcribed and analyzed for major themes. We identified an automated care management procedure of genetic testing that affects patient autonomy. The simple fact of having a written consent cannot justify a genetic test given the stakes associated with the results. We also suggest orienting practices toward a systemic approach using a multidisciplinary team or network to provide resources for dealing with uncertainties in interpreting results or situations that require additional technical or clinical skills and, if necessary, to allow for joint consultations with both a geneticist and a non-geneticist medical specialist.

## Introduction

Genetic data are by nature identifying, permanent, transmissible, and often predictive of health risk. They combine two characteristics not often found in other medical disciplines: the possibility of presymptomatic diagnosis (knowing a person’s genetic status before any clinical signs or symptoms appear) and the risk of passing it on to future generations. In view of this, to reduce the risk of discrimination and the slide toward eugenics, and to protect privacy and individual autonomy in decision making, the public authorities of many countries have regulated the use of genetic testing for example in preimplantation [1] or direct-to-consumer [2] genetic testing areas. In addition, European [3] and American [4] professional associations on human genetics regularly issue good practice recommendations. At the same time, because of new developments in medicine, biology, and genetics, and a revolution in technological tools that allow rapid mass whole-genome screening, genetic testing has become commonplace in all medical disciplines [5]. Genetic testing helps to identify the cause of a patient’s symptoms, putting an end to the search for a diagnosis [6], clarify the prognosis, offer precision treatments and develop treatment plans [7], defining a true genomic medicine [8]. This genetic diagnosis often helps provide social support to patients by referring them to appropriate patient organizations for help in coping with the isolation patients and their families often report feeling [9]. It also allows for reliable genetic counseling, i.e., better predicting the risks of other family members also being affected and those involved in family planning.

Medical geneticists have long played a central role in regulating access to genetic testing. This is both because of their academic training and daily practice and the regulations authorizing them to prescribe tests, provide pretest information, and return results. However, these days, all physicians prescribe genetic tests, even those not trained in genetics. This means that all physicians may find themselves in an unfamiliar situation: in addition to scientific matters about the relevance of prescribing a test and interpreting it, which can be complex, they must address the ethical and societal questions involved in deciding whether to test, and helping patients and families deal with the consequences of testing. Knowing how to inform patients of their genetic status, obtain consent, then manage the sharing of genetic information within a family requires skills that many have not learned. Therefore, it is essential that we consider and study how the prescription of genetic tests may change in many medical disciplines in which geneticists will no longer be the primary regulators. Several quantitative studies have already shown that non-geneticist healthcare professionals lack the necessary genetic knowledge or feel unprepared to deal with genetic test results [10, 11]. Likewise, non-geneticists consider handling family history [12, 13] or communicating genetic information to family members [14] to be difficult tasks. It has further been shown that inappropriate genetic counseling can result in harm to the patient and family [15]. Finally, the rapid increase in genetic technology also raises uncertainties about the data resulting from genome analysis and the consequences for patient health [16]. And yet, the development of genomic medicine, which has a role in promoting a health policy that is both predictive and personalized, is actively and officially supported in many countries such as England^1^ and France^2^. The population-wide use of DNA sequencing tools raises a number of medical, sociological, ethical, and legal issues to consider when expanding genetic testing [17]. Over and above challenges involving the training and knowledge of non-geneticist health professionals, it seems that patient support during genetic testing also requires developing new service organizations and different consultation modes. Therefore, to improve medical practices in the future, it is essential to have a thorough understanding of the difficulties and requirements for managing genetic testing, prescribing, and communicating results to patients, including performing tests and interpreting results.

The main objective of our study is to analyze how all of these factors interconnect in the trajectories of patients to whom genetic testing is offered: from the dynamics of recommending and prescribing genetic tests to communicating the results to patients and their families. A preliminary study conducted in similar fields [18] showed the importance of considering multiple dimensions: patient management, the organizational setup for providing pre- and post-test support (office visits, coordination with a laboratory, multidisciplinary cooperation between specialists with different areas of expertise), the regulatory dimension of laws and professional recommendations, and indirect impacts on relatives, transmission, and the clinical definition of a disease. Our study will contribute to describing the actors and practices and assessing the impact of offering genetic testing. This helps build pathways for improvement after the fact, based on an analysis of practices and organization of care, rather than predetermining general principles out of context. Furthermore, it will contribute to assessing how the developments in genetics impact medical training, cooperation between geneticist and non-geneticist professionals in the organization of care, and on the regulatory and ethical aspects.

## Methods

### Overview

This study used a qualitative methodology. We conducted focus groups with non-geneticist medical specialists to analyze professional practices and hear their questions and expectations. This method brings professionals together around a table to debate and exchange ideas on their practices and organization of care, with an external evaluator in charge of data collection. It allowed us to study the issues professionals face and how they respond to them (or not). It is recognized for allowing high-quality information saturation. This information was gathered by having professionals compare their practices and attitudes, taking note of debates, areas of complexity, areas for improvement, and expectations in different situations.

The protocol was developed by a multidisciplinary team with expertise in clinical genetics, public health, forensic medicine, health law, sociology, and ethics, as well as a representative from a patient organization. The ethics committee of Rennes University Hospital approved it on 13 June 2018 (ClinicalTrials.gov #NCT03572322).

### Participants and data collection

The survey collected data through focus groups. The scope was limited to four administrative regions in the western part of France (Brittany, Pays de la Loire, Centre-Val de Loire, and Normandy) and to two medical disciplines that commonly use genetic testing: endocrinology and neurology. This choice is supported by frequent genetic analyses that involve diagnostic and predictive testing as well as various care recommendations. On the one hand, in neurology, physicians are often confronted with progressive or even neurodegenerative diseases for which there is no cure (with Huntington’s disease being the prime example). On the other hand, endocrinology has many curative and preventive therapies available, particularly its oncologic (paragangliomas and pheochromocytomas) and functional (dysthyroidism or diabetes) branches.

Therefore, the physicians included had to specialize in either neurology or endocrinology as well as to prescribe genetic tests. The participants were contacted through a professional network of geneticists from each hospital in the four administrative regions chosen. The candidates were sent an email with a description of the project and an invitation to participate. Their consent was obtained, and their anonymity was guaranteed. No identifying professional or patient data appears in the collected data.

Interviews were conducted in focus groups, following an interview guide that allowed the only consultation observations to be clarified, nuanced, and enriched by injecting the physicians’ experiences and introducing discussions between the specialties. The interview guide was developed and validated after a preliminary study with two focus groups, each made up of three non-geneticist physicians who were not included in this study. It included the following topics: a presentation of each physician’s prescribing activity and trends, circumstances, prescribing process for genetic tests, knowledge of regulations, the ability to reflect on their activity and the process (See Supplementary data). Through these exchanges, the professionals learned about the practices, stress, dilemmas, and criteria of their colleagues in the other specialty.

Each focus group lasted an average of 90 min. The focus groups were conducted at the hospital, in an isolated conference room. During the focus groups, the two interviewers (a physician and a sociologist) took turns moderating, depending on the subject: while one led the discussion, the other took notes. When the exchange took more of a technical/medical turn, the physician stepped in to explain, and when it involved refining action logics or sources of stress in practice, the sociologist took over. Two information gathering techniques were used: note-taking during the discussion by the two interviewers (physician and sociologist), and audio recording of the entire interview with the participants’ agreement. In every case, the meeting was transcribed in full by an outside company independent of the investigators.

### Data analysis and processing

The data analysis involved the corpus of transcribed texts and observation notes from the focus groups. The analysis protocol is based on a comparative approach between two specialties and the processes they use. This approach corresponds to the methodological recommendation formulated by Timmermans and Tavory [19], which favors a position rooted in abductive analysis. Comparison enriches the scope of analysis of practices and differences, leading us to reflect on the nature of organizations, behaviors, and choices.

After the interview, the two investigators (physician and sociologist) shared their observations on the main takeaways, recurrent themes, and stresses noted in the prescribing process and for which actors. Surprises, unexpected situations, and anomalies led us to listen carefully during subsequent focus groups. Repeated reading of the materials, notations, and excerpts enriched the most meaningful themes and subthemes of the questions, problems, or solutions. The results were then presented and submitted to the multidisciplinary team for analysis, three times, to add content to the study and offer pathways for analysis. This is what allowed us to stabilize the content analysis framework.

## Results

### Sample characteristics

We conducted six focus groups of 21 physicians in 2018 and 2019, in six cities in the western part of France. This included 11 endocrinologists and 10 neurologists, all of whom prescribed genetic tests. The breakdown of the focus groups by specialty, sex, age, and experience of the physicians is shown in Table 1. Only one physician (a neurologist) reported having any specific initial training in genetics with completion of a PhD.

**Table 1:** Characteristics of enrolled physicians.

ID	Sex	Age	Genetics Training	Years in speciality	Number of tests per year
Neurologists					
A	Male	32	No	3	3–5
A	Male	–	No	–	60
B	Female	48	No	20	10
B	Male	65	No	35	20
C	Female	49	No	24	3–5
D	Female	43	No	12	70
D	Male	51	Yes	20	100
E	Female	47	No	15	30
E	Female	40	No	12	10
F	Male	46	No	15	75
Endocrinologists					
A	Female	38	No	9	10
A	Female	43	No	15	10
B	Female	–	No	10	40
C	Female	35	No	8	5–10
C	Female	–	No	10	3–5
D	Male	43	No	19	20
E	Female	42	No	14	30
E	Female	37	No	9	50
F	Male	–	No	25	5–10
F	Female	38	No	8	5–10
F	Male	63	No	34	20

### Recurrent themes

We highlight the three following points that emerged from the interviews: (I) a segmented practice rooted in the specific characteristics of each disease, (II) difficult situations that create stress due to limited skills, and (III) strategies for coping with this stress. The following results are divided into these three categories.

#### (I) A segmented practice rooted in the specific characteristics of each disease

##### In endocrinology, genetic testing has become commonplace and automated

In the field of endocrinology, practices were found to vary between oncologic endocrinology and functional endocrinology. In oncologic endocrinology, with regard to the nature of diseases, its characterization, and related protocols, the use of genetic testing has become automatic. Thus, in the following excerpt:

“*For rare diseases such as pheochromocytomas and paragangliomas, new genes are constantly being discovered. That contributes to the characterization of these diseases, to better understanding their specific characteristics, and thus perhaps result in new leads, to therapeutic targets, so it’s both to better understand these diseases, better treatment, and for family screening, too, so that’s routine and not only based on clinical signs and symptoms, and that helps better characterize knowledge. For pheochromocytomas and paragangliomas, it would be illegal not to test. It is related to treatment, monitoring in any case, and quite close monitoring, and, therefore, ordering certain more reliable tests for such a mutation is a lost opportunity if it is not known that a patient has that disease*.” Focus group B.

What is interesting in the above excerpt is the emphasis placed on the temporal relationship and the similarity of the activities. The surgery is affected by a change in the course of the disease: what focused selective attention on a highly delicate treatment decision is only temporary. Routine monitoring leads to steering the disease toward a genetic scenario. The testing indication is described as almost routine when the patient is admitted to a hospital for surgery. The account given by this endocrinologist, who focuses on monitoring endocrine cancers, illustrates the connection between the moment of surgery, hospitalization, and the testing scenario for genetic predisposition.

*“Pheochromocytomas and paragangliomas are usually genetically diagnosed as part of a preoperative assessment, so early as well. Patients are hospitalized in preparation for surgery, and that’s part of the package, in fact, they come and have a genetic test during their hospital stay. They are usually warned of this ahead of time, but not always, because sometimes in the rush to surgery we are crunched for time.”* Focus group E.

The combination of hospitalization, an endocrinology assessment and sample-taking all fall under a sort of windfall effect. In other words, the procedure leading from a cancerous pathology to hospitalization to assessment, sends a signal to order genetic tests. Like a cascade, a series of tasks leads to the tests being ordered. This windfall effect is produced when the spatial and temporal environments positively influence the recommended prescription within the framework of a medical reference without it being planned in advance. Thus, in the following excerpt, which concerns a surgical intervention:

“*There are patients who are first discovered on the day they come. For example, they have an office visit with an ENT, the ENT discovers the paraganglioma, so they are referred to us for an extension study to see if there is any elsewhere, and we take the opportunity to do some genetic tests. So, we discover them, let’s say, the day they arrive during hospitalization. So, they sign the consent form on the day they’re informed, there is no time afterwards and the family tree is filled out, or, actually, medical students fill out the family tree, and the form is filled out right then*.” Focus group A.

The predictable pace of chronic diseases and consideration of the major impacts, knowledge of the vital prognosis, and the therapeutic decision lead to an organizational setup that automatically triggers genetic testing. Therefore, we suggest standardized prescribing, a way of automating procedures. For tumors, the genetic hypothesis and therapeutic recommendations seem to be the two identifying factors of another emerging medical logic.

In functional endocrine disorders, genetic testing is begun more to improve diagnostic precision using biomolecular data.

“*In the functional part, what I was telling you about the thyroid is more aimed at understanding what happens and especially avoiding pushing testing further if there is a certain genetic result. We know that the thyroid assay is always going to stay like that and so it closes the debate and avoids additional tests and unnecessary treatments…while sometimes we….we find ourselves doing genetics, that is correcting the endocrinological diagnosis using genetics*.” Focus group D.

In summary, we can identify what unites the two groups of endocrinological diseases beyond their different trajectories and forms of expressivity. An emphasis is placed on early testing for treatment and family screening, and on the genetic causality of expressions.

##### In neurology, given the lack of therapeutic tools, a process of “micro announcements”

In preexisting hereditary neurological diseases, there are two situations where testing may be indicated: [1] symptomatic testing to confirm a diagnosis, [2] presymptomatic testing when there is a significant risk that symptoms of a progressive disease will appear. For presymptomatic tests, the channels are defined as “systematized” (or even regulated in some countries such as France) with geneticists in charge, while the channel for symptomatic patients suspected of having a degenerative neurological disease (such as Huntington’s or cerebellar ataxia) goes through a neurologist first. This channel begins with questions about family history, which is a recurrent theme. Thus, this neurologist, who is accustomed to these practices said:

“*How is it practiced? Studying the family history is part of the routine intake, so what is your personal family history? Today we have more and more diseases where that is part of it regardless of the mode of entry for studying the family history*.” Focus group C.

Having two consultations generally slows the prescription channel, allowing time for shared consideration between the clinician and the patient. In the excerpt below, we feel the doubts and the questions, which spill over only from the constraints of the agenda and the coordination between the prescriber and the laboratory, as the following account illustrates:

“*It’s done in several steps overall, that is, for me, in any case, since anyway I never manage to get it all done during one visit…basically, when I see people for whom I didn’t necessarily anticipate any reason for taking a sample. In any case, I see them to organize sampling, so… generally they already have some idea what’s going on, at least at the first visit they have an idea of what’s going to be done, what can be done, what we’re looking for, what it’s going to provide… in any case, it’s at that moment that I explain all that, so that afterwards I ask them to think about it and get back to me to let me know if they are okay with the samples being taken. If they are OK with the samples I’m organizing for the second visit where I previously anticipated filling out very long forms and consents to prepare, etc., and I made an appointment with the sampling center… so, finally, there is a time lapse between the moment the hypothesis is raised and the sample is taken, and then explaining what we’re going to look for, and, finally, the agreement and the sample taking*.” Focus group B.

Finally, at the time the results are transmitted showing genetic abnormalities, the exchanges concern mainly the important impacts of this revelation. We noted the emphasis placed on the moment chosen to apply the disease markers and make micro announcements beforehand. In this excerpt, the hospitalization, the assessment, and the consultation are defined and conducted with a view toward increasing the value of a specific, crucial exam, particularly during the trajectory of the affected subject:

“*All the same, I prefer making this important announcement in an outpatient facility because I don’t think it’s a trivial thing. That also gives us the time to properly fill out the family tree, clinical information for the laboratory… to give the patient enough time to absorb the announcement, that’s how I see it, at the time of a hospitalization…*

*I1: Just so I understand, what does “absorb the announcement” mean for the person?*

*XX: So that we aren’t accused later of having done genetic testing that may be requested a bit hastily without properly informing the patient so that this is a significant event in the course of the patient’s disease… That’s how I see it, but it’s my personal opinion. It is not a trivial test…*” Focus group A.

In neurology, the frame of reference gives us a glimpse of the increased systematization of indications: the family history and story written down in a family tree, selective attention to temporality (age, early screening), and risk (susceptibility and predisposition). The tone of the exchanges was marked by two concerns: allowing enough time for the patient and family to absorb the information; the hospital setting and long appointment times as vehicles for dramatizing this genetic research scenario. At this stage, the reflective work is done by the physicians and patients. Thus, beforehand, the discourse and practices give free rein to the exchanges, information learning, and explanations on this suspected disease and its probable hereditary nature.

#### (II) Difficult situations that create stress due to limited skills

The data from the focus groups show sources of stress and dilemmas when the follow-up procedures and treatment plans for the two specialties lead to ordering a genetic test. The analysis shows that this intersection between care by a specialist and management of genetic testing reveals tensions that clinical physicians face with regard to their reflective work. In both specialties investigated in our study, the main sources of stress for professionals are as follows: [1] coordination and cooperation between specialists and organization of care as a team; [2] impacts on the patient’s genetic status and on the family, put to the test during this sequence where a disease and a hereditary explanation cross; [3] the enigmas posed by the diverse effects of genetic research (variants of unknown significance) that raise uncertainties at the frontier between a medical specialty and genetics.

##### A hybrid announcement meeting—at the interface of medical specialties and genetics

During the consultation to announce the test results, the specialists’ questions concerned the qualifications and skills they needed in practice, such as the overlap between endocrine, neurological, and genetic knowledge, the limits of legitimacy when explaining a diagnosis or prognosis to the person or family and the ability to use appropriate, correct, and positive terms. The interviewees expressed this stress as feeling “uncomfortable” about their inability to handle the patients’ and parents’ questions, since they felt they lacked the necessary genetic knowledge.

A first tension can be seen when issues such as diagnosis, prognosis, and genetic counseling are raised entirely or partly by the geneticist. Both neurologists and endocrinologists mentioned their limited skills in that area. This neurologist frankly admitted her concerns about having the skills and appearing professional, and the risk that a worried patient might not trust her:

*“Frankly, I don’t always feel comfortable making this announcement, and I would like…. that’s where I would really like us to imagine mixed consultations, if it were possible. It’s hard for me to answer all the questions and maybe I still don’t always have the right terms, in any case, about the phenomena of anticipation, penetrance, phenotype, all that sometimes…. even if it has to do with diseases that I’m familiar with, there are still new questions I have never been asked. People can ask questions about genetics that are always rather troubling, such as about paternal and maternal transmission. There are many, many of them and sometimes it throws me off track a bit. Then I’m in trouble!”* Focus group B.

This other neurologist tells of her social and professional indecision during complex and undetermined consultations. She sees herself as comfortable with known diseases, but feels uneasy with advances in genetics when she knows she is (legally) bound to explain the results.

*“I am much less comfortable because I have had feedback from patients who struggled a bit with their decision and who, between the time I mentioned the possibility because they themselves, a 35-year-old patient with Parkinson’s disease, can’t help wondering about the genetic potential of his disease. And I can’t always support patients the way I would like because I don’t have the genetic tools to do so. I don’t have a geneticist’s knowledge or experience that would allow me to reassure the patient about whether to go ahead or not and to counsel her about all the family issues it involves*.” Focus group E.

The complexity of pathological situations is such that the division of labor between non-geneticist medical specialists and geneticists remains essential. During consultations, when the announcement and treatment phases take a rather genetic turn, the specialist generally refers the patient to a genetic counselor.

##### A mixed consultation to provide information and emotional support in the face of a hereditary disease

A second tension, keenly felt by practitioners, accompanies the information delivered with the genetic test results, then communicating them to the patient and the family. This tension is common in medical practice. However, during this consultation we also heard from practitioners in terms of the need to demonstrate the importance of genetic testing not only to the individual but to the family. That which is a delicate matter for oneself in a chronic disease, through heredity, becomes delicate in another way, in one’s intimate and social relations, with one’s existing biological family, and family to come—that of the next generation. In rare cases, family configuration leads to an initial refusal of genetic testing, precisely because of the fear of revelations about the familial, hereditary nature of a given disease. This endocrinologist reminds us:

“*It’s a family where we highly suspected a genetic diagnosis in a patient who refused to do a genetic survey, and we knew that if the children were affected, we could screen them early and avoid sudden death or that sort of thing. It is more in the refusal to do the genetic survey that that poses a problem, in fact. Sometimes it’s hard to find arguments to change their minds…*” Focus group C.

With this type of stress, the interviews remind us of the value of a well-reasoned response to the parents’ questions. That is the case for this endocrinologist:

“*I think that when genetic results are announced, we have to announce the penetrance of the disease. We have to announce the diagnostic methods, the follow-up methods, as well as the current treatments, which may not be those that will be offered tomorrow because of the disease… so we don’t know. We have to be able to explain all that because the big question that keeps coming up is, after all, ‘and my children? What risks are there to my children? Does it skip generations?’ These are the three questions that come up repeatedly*.” Focus group E.

More generally, a personalized diagnosis requires sharing the information within families, and this can create problems. Once a precise diagnosis is confirmed in an affected person, genetic counseling allows parents or relatives to consider a presymptomatic or prenatal diagnosis. As there is no treatment for many neurological diseases, and therefore no medical benefit to the patient, an unfavorable result may lead patients to change their reproductive plans. Finally, the question of family screening can disrupt deeply rooted images of parenthood. The term screening simultaneously impacts the subject’s reality and the family ecosystem: if screening is done and the result is positive, the disease becomes a matter for the whole family, not just one specific person. Again, the arguments for early testing and premature treatment, as justifiable as they are, medically speaking, do not eliminate the fact that the screening results deeply alter the family’s genetic map. Thus, an endocrinologist tells us:

“*Some children that can be operated on as early as six months for the thyroid when we know they are transferred, because the cancer may be there starting at six months, so that has a direct implication. Then, it can be recommended later, but I am concerned about several patients for the screening of their children. (…) Finally, I find that very hard to manage to explain because this father who, on top of everything, has been through so much, and even has metastatic cancer, is not going to get his kids screened. I find that extremely hard to understand and to accept from my point of view. After all, I’m not in his place but I find that…… it’s the whole discussion of the problem of family screening… but it’s hard! When it’s brothers and sisters and all that, it bothers me a bit less in adults, but they are responsible for their children, and they shouldn’t do that to children who aren’t able to judge for themselves…!”* Focus group A.

The physicians interviewed found it challenging to find the delicate dividing line between announcing a disease and announcing the genetic nature of the disease. Deciding to perform a genetic test is a source of hesitation: the idea of communicating the information may be refused. Even if the information is accurate and noninvasive, well-reasoned, and discussed, it may create ambivalence leading to denial, and a refusal to find out. The reasonable promise of a better future for oneself and one’s family is countered by the anxiety of an uncertain destiny. The suggestion of a genetic test may lead to delaying a decision. The sense of the future is a source of uncertainty: hopes for children and grandchildren are called into question. What use is it to be given a genetic label? As this endocrinologist says:

*“I have someone whose mom died, and her adolescent child was screened later, I think that… it’s a common reaction to stick your head in the sand and say no, the mutation couldn’t have been passed on to her child…*

*I1: Do you mean that in spite of the fact that you gave them that information, they were still in denial?*

*XX: It’s true that we are quite often confronted with that, and it’s a tricky thing to say that there is a person who could be screened, who could be treated, who could be cured, and who may now develop metastatic cancer because she was not able to receive care. And I have a patient whose daughters got screened, and they waited until the first children were adults before they started screening. It took them 20 years before they started screening their children.”* Focus group A.

Once again, in this excerpt, the complex question of the link between an individual’s genetic profile, family recomposition, and projected timeline are expressed.

##### Enigmas: ambiguous results and new situations

Some genetic results contain information that is not unambiguous with regard to current knowledge. It requires a wealth of knowledge to obtain clearer information that can confirm whether there is a pathological role related to the phenotype observed. In that regard, this type of stress is the subject of questions and mitigation strategies for practitioners. Some physicians noted that the management of these variants of unknown significance is part of medical practice and gives rise to increased cooperation with clinical geneticists, or laboratory researchers. This endocrinologist describes this dialog about the degree of “relevance” (significance) or “pathogenicity” of the variations of a given genome:

*“We also have this problem during our monthly meetings with the molecular biology [department] of knowing if what we found as a variant is relevant or not, so it’s a daily problem, as well. We encounter it in particular cases for deformities or combinations of symptoms that we feel have a genetic component, a little endocrinological, but not only that*.” Focus group D.

Navigation between the expected and unexpected is something specialists deal with in their work every day, along with uncertainties that arise, leading professionals to debate their meanings, but also what information to deliver, the conversation with the patient and family when the prognosis is vague. This neurologist tells us about a case:

“*In fact, we have a case of Huntington’s disease where neither parent is symptomatic. Or there’s a symptomatic parent on one side, but we find that the mutation comes from the other side of the family! Or once there was a situation where neither of the two parents was symptomatic and, in fact, it was because there was an intermediate allele…*” Focus group D.

The interviewees strongly felt that ambiguous results should be shared with the family. The practitioners said that they were avoiding the issue while awaiting new data and knowledge on the case, as this endocrinologist tells us:

“*We keep it a little vague, anyway, a patient who has a paraganglioma, we don’t know if it’s pathogenic or not. The parents were studied for it. I’m going to do my pathology follow-up and I make it a little abstract while waiting for the science to evolve. Afterwards, I had a mutation study done on an adolescent in the presence of the parents, she agreed, and we had the parents sign the consent form*.” Focus group A.

We could mention other specific cases, giving rise to moderate expressions of the disease that are uninterpretable.

“*Our problem… it’s going to be rather… but like everyone, for example, in a disease considered to be recessive, we find something heterozygous and wonder whether we’ve found a “light” form of heterozygote, or whether it has nothing to do with it and is a pathogenic variant but one that isn’t expressed because it would have to be homozygous, so we often have this sort of question that we’re not going to have trouble interpreting*.” Neurologist, Focus group D.

Returning results to patients and the family is touchy since it is based on the findings of biomedical entities that are uncomfortable with interpreting incomplete statements.

#### (III) Strategies for coping with stresses

The fact that they do not deal with these situations and the emotional climate often leads practitioners to ask for help. To deal with these stresses, these non-geneticist physicians mentioned two nonexclusive strategies:

##### Consultations with a geneticist and a non-geneticist physician

For reasons of technical knowledge or even uncomfortable situations that destabilize the physician’s position as an “expert,” non-geneticist physicians said many times that they would prefer to conduct these consultations together with a geneticist.

*“…frankly, I don’t always feel comfortable having to make that announcement, and I would like… in that case, I would really like us to imagine, if it were possible, mixed consultations….”* Neurologist, Focus group B.

*“That is really what makes sense in returning the results, ideally… the possibility of doing a mixed consultation (…), the geneticist and the neurologist, or the endocrinologist, or with the family to be able to explain the thing more relevantly rather than just doing one then the other and not together.”* Neurologist, Focus group B.

They are hard to arrange because of the lack of geneticists available on site, the geographic distance between teams at the same hospital, or the rarity of the disease, which explains the apparent difficulty in establishing a specific organization. These non-geneticist practitioners expressed a desire for more collaboration, confirmed by their wish not to entrust the announcement of a genetic disease to geneticists alone, especially because they need to be part of the patient’s overall treatment, which is not really in the area of this specialist alone:

*“The geneticist alone won’t do much, if it’s just a matter of pressing a button and giving a result, anyone can do that after a while. The problem is merging it with the clinical manifestations. And as soon as one of the two is missing, the genetics won’t make any sense!”* Endocrinologist, Focus group D.

##### The need for collegiality and inter-professionalism to guide choices and decisions

The sources of stress reported show how concerned the physicians involved are. How are these difficult situations discussed among professionals? By means of collegiality, which is the vehicle for professional reflexivity. Three sources of stress are alleviated in staff meetings combining physicians, genetic counselors, geneticist clinicians, and psychologists *with* and *through* case files, borderline cases, and results of uncertain significance.

These two excerpts from endocrinologists exemplify this practice of asking questions, sharing resources, and making requests within the debate arenas, often prior to the consultation where it will be announced:

*“That’s just the problem now, of the variants of uncertain significance with the development of the microarray, so, afterwards, it’s a dialog with the laboratory, and often what comes back is, the laboratory result is quite clear with regard to the pheochromocytomas and paragangliomas, and voilà… that’s sort of what we have to do… a fairly clear finding either can’t be envisioned, or we can’t envision the family screening in patients or we’d have to say “thanks for sending us such and such a sample so we can complete the studies…”* Focus group B.

*“We started with the targeted test and now we’re on microarrays. So, they don’t give us a result as long as there’s no coordination, as long as we haven’t had our endocrinology molecular biology staff meeting. So, from that moment on—at the time of the staff meeting, we agree on what to answer, is the variant we found, is it pathogenic, yes or no? And when we don’t know, to tell the patient we don’t know, and so on (…) But in that case, we have to stick with the clinical findings. We have a pathology, symptoms, a series of signs, if the result is completely inconsistent with that, we’re not necessarily going to tell the patient…”* Focus group D.

Finally, the difficulties generated by the very nature of genetic testing are less likely to encourage physicians to pursue additional training than to ask for assistance and cooperation from their geneticist colleagues.

## Discussion

With regard to genomic medicine, there is a significant gap between medical specialists who may occasionally have to deal with genetic testing and medical geneticists for whom genetic testing is part of their daily practice. Thus, over years of experience, geneticists have developed a discourse, tools, and a network of national and international partnerships, i.e., a whole environment that allows them to manage the test recommendation, perform the test and announce the results. This includes managing the uncertainties [19] and difficulties inherent in this discipline: interpreting genetic variants whose pathogenic nature is rarely obvious [20], supporting the patient, sharing information with the family (especially when there are family difficulties or conflicts), and handling requests for prenatal or preimplantation diagnosis. Non-geneticist medical specialists lack this expertise, but they do have solid experience in the diagnosis, prognosis, and treatment of disease. In this environment, prescribing genetic tests is an important new tool for diagnosis and therapeutic guidance, but it is done gradually, along with all the other tasks the prescriber has to manage during the consultation. Today, it is no longer a matter of knowing whether a non-geneticist physician is able to prescribe a test or not; the situation and changes in practices mean that genetics are available outside of genetics services and will be in the future. The important thing is to understand what is at stake and how professionals deal with it.

Our study sheds light on it through the prism of a qualitative study with professionals from two medical specialties: (i) A first key to understanding prescribing partly has to do with the very nature of the medical specialty (neurology or endocrinology). We have noticed a change over time in relation to the gradual emergence of genetic tools in clinical practice and in the role or impact of genetic data on the patients’ treatment. Neurologists have a longer-standing interest in genetic testing since the gene responsible for Huntington’s disease was identified in 1993. There is much more familiarity with genetic testing among neurologists. As many practitioners pointed out in the focus groups, neurology has a sort of tradition of using genetics through family history. Neurology covers serious and debilitating degenerative diseases and many times can offer only palliative therapy. Thus, genetic testing is often done to guide diagnosis, to give a name to a disease, and to clarify the prognosis. It also possibly provides genetic counseling to the family. In endocrinology, on the other hand, it seems that tests are usually prescribed to guide a therapeutic decision. It is used much less often, and endocrinologists are slower to turn to genetic testing. (ii) A second key to understanding concerns the impact of the organization of care and specifically the structural configuration, which appears similar in the two specialties. Between a facility labeled as a reference center (effect on drawing patients away from the region, sizeable patient lists), and more fragile ones in a mid-sized hospital, the impacts are felt on learning to use genetic testing on one hand, and regulation of ways to coordinate with laboratories and the network, on the other. (iii) The third key to understanding is similar in both specialties: it concerns the generational effect, with a notable difference between young professionals and older ones. The younger ones have at least been made aware of genetics during their academic training as an opportunity and a body of resources. Older neurologists, although more experienced in many aspects of their discipline, have had to educate themselves and adapt to the connection between a chronic disease and a family tree. The oldest clinicians seem more reluctant and cautious with this technology.

Two problems emerged from our qualitative study that should be taken into account in physician training and in their genetic testing practices:

Either because a practice adopts routine procedures (as part of a protocol) in endocrinology, or in the context of a neurological disease that leads to vulnerability and limited cognition, it seems there is a risk of genetic tests being prescribed without a full informed consent process, raising the issue of failure to respect the patient’s decision-making autonomy. At the heart of this matter of “*information process”* lies a major question, equally complex, which professionals should make part of the care relationship: knowing how to explain the probabilistic nature of a result, its level of uncertainty and the familial stakes. The physicians need to know how to balance knowledge, which is often probabilistic, from prognostic and predictive genetic biomarkers with the impact on *individual choice*, informed if possible. On the subject of ethics, it is widely agreed that subjects must be able to make autonomous choices about the things that concern them. In concrete terms in France, this means obtaining a signed, written consent form after the patient has been clearly, honestly, and appropriately informed of the issues involved in genetic testing. This written consent is meaningful only if it has been clearly explained and understood, which depends on the methods and skills that professionals use in their practice. Beyond the bioethics rhetoric of “patient-centered process”, the reality of medical consultations reveals the complexity involved in truly respecting patient autonomy. There is a danger of an informative illusion when the practitioner tries to deliver purely scientific information without taking the patient’s emotional reality into account. We should also examine how patient intake, information time, question time, and especially reflection time, are organized and structured. We showed that the combination of organizational constraints and rules of medical follow-up play a major role in standardizing test prescribing practices, and thus changing the norms of autonomy governing the exchange of information between specialist and patient. Specialized literature in human and social sciences has long alerted us to the frequent confusion between knowledge and understanding, between data and results, and between information and interpretation, as well as the ambivalence [21] of people torn between the quest for knowledge and making sense of what happens to them. Actually, the political, legal, and ethical plan governing the information and signed consent process defines a restrictive system in France. But beyond the restrictions imposed, it is important to look at how to organize care in such a way as to best meet the expectations and needs for understanding of patients facing endocrinological and neurological diseases in the future. The quality of the information provided, regardless of whether the patient agrees for himself, is also worth questioning with regard to the stakes for the family. In addition to the index subject, the research process and genetic results expand the scope of the disease to the family and reconfigure family relationships. Thus, the family’s destiny is thoroughly “geneticized” in the guise of genetic predisposition to a given disease, embodied in a specific patient. As, in France, a strong regulatory and legal framework governs the sharing of genetic information with family members [22], the fact that all professionals are familiar with it and even integrate it into their practice is important.

The request for increased collaboration between geneticists and other medical specialists clearly demanded during our study—and ways to arrange areas for them to meet, particularly staff meetings and joint consultations—is not new. However, it is now time to implement it. Having professionals work together with their separate cultures is part of a medium-term objective. In a recent article [23], the authors used a similar qualitative method that included 11 primary care physicians and 9 cardiologists in the United States in semi-structured interviews, after a short, one-hour training session, and before returning whole genome sequencing (WGS) results to their patients. The emerging themes were their concerns about preparedness and motivations for developing proficiencies. Cardiologists, in particular, felt an obligation to respond competently to cardiac-related results, yet they could miss information as the French specialists did. Thus, most of the physicians wanted geneticists to be more involved in providing information and returning results. In another qualitative study on sharing genetic information with family members conducted in France by means of semi-structured interviews [24] with physicians specialized in genetics, these physicians regretted the lack of cooperation with non-geneticist medical specialists. From the point of view of medical geneticists, the time spent explaining the nature and benefits of genetic testing is crucial and appears to be a key factor in successfully communicating information to the family.

In conclusion, our study suggests steering new practices in genomic medicine toward a systemic approach using multidisciplinary teams and networks. These teams and networks would include specialists in organ systems and in clinical and laboratory genetics to serve as a treasure trove of resources for dealing with uncertainty and with situations with technical or clinical limitations. Consultations would ideally be conducted with both a geneticist and a non-geneticist medical specialist. We also have to find the fine balance between procedural constraints, shortened time frames, incitement to do genetic testing, and patient autonomy.

### Limitations and research recommendations

As this is a qualitative study with a small nonrandom sample size, the findings cannot be generalized. However, the authors claim that the findings may be pertinent to other similar medical specialties in different European countries. These data must be supplemented by a similar study with patients who receive genetic testing to evaluate their perception of how their autonomy is respected.

## Supplementary information

Interview Guide

## Footnotes

1 https://www.genomicsengland.co.uk/

2 https://pfmg2025.aviesan.fr/en/
