# Supplementary material for: How do non-geneticist physicians deal with genetic tests? A qualitative analysis
Source: Eur J Hum Genet. 2021 Apr 28;30(3):320–31. doi: 10.1038/s41431-021-00884-z (PMC8904857; doi:10.1038/s41431-021-00884-z)
Supplement: Supplementary file 1 — Interview Guide [file 41431_2021_884_MOESM1_ESM.docx]

***Interview Guide for physicians (focus groups)***

1. ***Presentation of each physician's prescribing activity and trends, circumstances***

- Describe your academic career and your experience in speciality.
- Describe your academic structure your work in
- Data about genetic tests: How many tests a year and evolution

1. ***Prescribing process for genetic tests***

- Which are the circumstances to prescribe a genetic test?
- Describe step by step how is it managed (information – consent – sample)
- Describe how the patient is informed about the result: duration, patient alone / family…
- Have you come across any specific issues? Under-18 / adult, not able to give a consent, uncertainties about clinical diagnosis or genetic variation, unsolicited findings, family communication…
- What are the means to solve the problems? Databases, staff meetings, geneticists… Would you consider some consultations with a geneticist?

1. ***Knowledge of regulations***

- What is the legal framework (in France)?
- Do you know some professional recommendations?
- How would you consider the term “seriousness”?
- Is the legal framework justified in your day-to-day practice?

1. **T*he ability to reflect on their activity and the process***

- What is the medical utility (appropriate, available, convenient)?
- Does the genetic test trigger some changes in your job? Nosography, diagnostic and treatment guidelines, family communication…
- What would you suggest to improve your knowledges / practices?
- Have you got some comments listening to your colleagues?
